# Perioperative outcomes of neoadjuvant chemotherapy plus camrelizumab versus neoadjuvant chemotherapy plus tislelizumab for locally advanced esophageal squamous cell cancer: a real-world retrospective study

**DOI:** 10.3389/fimmu.2025.1544739

**Published:** 2025-08-21

**Authors:** Qi Zhao, Yusen Yuan, Tongxin Xu, Ningning Yan, Fei Li, Juntao Lu, Ming He, Zhaoyang Yan

**Affiliations:** ^1^ Department of Thoracic Surgery, the Fourth Hospital of Hebei Medical University, Shijiazhuang, Hebei, China; ^2^ Department of CT&MRI, the Fourth Hospital of Hebei Medical University, Shijiazhuang, Hebei, China; ^3^ Department of Thoracic Surgery, the Second Hospital of Hebei Medical University, Shijiazhuang, Hebei, China; ^4^ Laboratory of Pathology, Hebei Cancer Institute, the Fourth Hospital of Hebei Medical University, Shijiazhuang, Hebei, China

**Keywords:** esophageal squamous cell carcinoma, neoadjuvant therapy, tislelizumab, camrelizumab, immunotherapy, esophagectomy, efficacy, safety

## Abstract

**Background:**

While neoadjuvant chemoimmunotherapy shows promise for locally advanced esophageal squamous cell carcinoma (ESCC), optimal regimen selection remains challenging. This study compares perioperative outcomes between camrelizumab- and tislelizumab-based neoadjuvant chemoimmunotherapy in ESCC.

**Methods:**

We conducted a retrospective analysis of 209 clinical stage II-IVA ESCC patients treated at Hebei Medical University Fourth Hospital (October 2020-December 2023) who underwent neoadjuvant chemoimmunotherapy (camrelizumab, n=119; tislelizumab, n=90) followed by esophagectomy.

**Results:**

Comparable pathological responses were observed between groups: pathological complete response (31.1% vs 30.3%, P=1.00), major pathological response (44.4% vs 42.9%, P=0.89), and pathological downstaging (67.8% vs 73.9%, P=0.36). Perioperative complication rates, including hematologic toxicities, immune-related adverse events, and surgical complications, were similar (all P>0.05). The tislelizumab group demonstrated significantly lower unplanned ICU transfer rates (P=0.04), while operative parameters (duration, blood loss, R0 resection) showed no differences.

**Conclusion:**

Tislelizumab-based chemoimmunotherapy demonstrates comparable efficacy and safety to camrelizumab-based regimens, potentially representing a viable neoadjuvant option for locally advanced ESCC.

## Introduction

Esophageal cancer ranks seventh in incidence and sixth as an overall cause of mortality worldwide (1). The incidence of esophageal cancer exhibits significant regional variations. In China, esophageal squamous cell carcinoma (ESCC) accounts for over 90% of all cases of esophageal cancer (2). Surgery remains the standard treatment for locally advanced ESCC. However, it has been reported that approximately 33% of patients with ESCC who undergo surgery alone experience local recurrence and distant metastasis (3, 4). Since the publication of the results of the CROSS, NEOCRTEC5010, and JCOG9907 trials, neoadjuvant therapy has emerged as the first-line treatment option for locally advanced ESCC (5–7). In East Asia, thoracic surgeons tend to favor neoadjuvant chemotherapy over neoadjuvant chemoradiotherapy (8, 9).

The remarkable therapeutic efficacy of immunotherapy (including programmed cell death-ligand 1 [PD-L1] and programmed cell death protein-1 [PD-1] inhibitors) in advanced esophageal cancer has prompted numerous researchers to investigate its potential application as neoadjuvant therapy (10). A recent multicenter retrospective study demonstrated that patients with locally advanced ESCC who received neoadjuvant chemoimmunotherapy achieved superior 2-year overall survival (OS) and disease-free survival (DFS) outcomes compared to those treated with neoadjuvant chemoradiotherapy alone (11). The open-label, randomized Phase III ESCORT-NEO/NCCES01 trial showed that neoadjuvant chemotherapy plus camrelizumab is a safe treatment option for locally advanced ESCC and can enhance the pathological complete response (pCR) rate (12). Tislelizumab, a PD-1 inhibitor, has demonstrated promising efficacy and safety in the treatment of advanced ESCC, as evidenced by the RATIONALE-302 study (13). The TD-NICE study also found that tislelizumab combined with chemotherapy as neoadjuvant therapy improved the major pathological response (MPR), pCR, and R0 resection rates in resectable ESCC while maintaining an acceptable level of tolerability (14). In clinical practice, both of these immunotherapeutic agents are used in combination with neoadjuvant chemotherapy for locally advanced ESCC. However, direct comparisons of the therapeutic efficacy of these two agents during the perioperative period are currently limited.

In this study, we compared the perioperative efficacy and safety of neoadjuvant chemotherapy plus camrelizumab with that of neoadjuvant chemotherapy plus tislelizumab in patients with locally advanced ESCC.

## Methods

### Inclusion and exclusion criteria

This study had a single-center, retrospective real-world design and included patients with resectable locally advanced ESCC who received neoadjuvant chemotherapy plus camrelizumab or tislelizumab followed by esophagectomy between October 2020 and December 2023 at the Fourth Hospital of Hebei Medical University. The primary objective of this study was to evaluate and compare the therapeutic efficacy and safety outcomes between the two neoadjuvant treatment strategies in the perioperative setting. Data for these patients were collected from the electronic medical records. The inclusion criteria were as follows: pathological diagnosis of ESCC; resectable clinical stage II–IVA ESCC according to the American Joint Committee on Cancer 8^th^ edition TNM staging system; received a complete cycle of neoadjuvant chemotherapy combined with camrelizumab or tislelizumab; and resection performed. Patients with cervical esophageal cancer, T4b disease, other tumors, or incomplete medical records were excluded.This study was conducted per the Declaration of Helsinki (as revised in 2013).

### Staging and treatment

The patients underwent baseline staging using contrast-enhanced thoracoabdominal computed tomography, endoscopic ultrasound, and cervical lymph node ultrasound. Positron emission tomography–computed tomography was used for tumor staging when necessary. Clinical and pathological staging was performed using the American Joint Committee on Cancer 8th edition TNM staging system. Before surgical resection, all patients received 1–4 cycles of immunotherapy (camrelizumab 200 mg or tislelizumab 200 mg) and chemotherapy (platinum-based agent and albumin-bound paclitaxel/docetaxel) every 3–4 weeks. The specific chemotherapy regimens were as follows: cisplatin (75 mg/m² D1) or carboplatin (AUC=5 D1) combined with albumin bound paclitaxel (260 mg/m² D1); cisplatin (75 mg/m² D1) or carboplatin (AUC=5 D1) combined with docetaxel (75 mg/m² D1). Surgical procedures included McKeown and Ivor Lewis esophagectomy. Two-field lymph node dissection was routinely performed, with three-field lymph node dissection applied only when cervical lymph node metastasis was considered. All procedures were performed by high-volume surgeons with >5 years of specialization in this field. The criteria for unplanned postoperative ICU transfer included severe pneumonia, respiratory failure, significant arrhythmias, septic shock, and other life-threatening complications.

### Outcome measures

A pCR was defined as the absence of evidence of residual tumor cells at the primary site in the surgical specimen and in the resected lymph nodes. An MPR was defined as having less than 10% viable residual tumor cells in the resection specimen. The tumor regression grade (TRG) was determined as follows: TRG 0, no visible viable cancer cells; TRG 1, single cells or rare small groups of cancer cells; TRG 2, residual cancer cells present, indicating significant tumor regression but extending beyond single cells or rare small groups; and TRG 3, a large number of residual cancer cells without obvious tumor regression. Operation time was calculated as the time between incision and wound closure.

### Statistical analysis

As this was a retrospective study, our sample size was limited to all available patients who satisfied the inclusion criteria. Categorical variables are expressed as the count and percentage and were compared between groups using the chi-squared test or Fisher’s exact test. Continuous variables are shown as the median and range and were compared between groups using the Wilcoxon rank sum test. All statistical analyses were performed using STATA version 15.0 software (StataCorp LLC, College Station, TX, USA). A two-sided *P*-value of <0.05 was considered statistically significant. A *post-hoc* power calculation (two-sided α=0.05, power=80%, β=0.20) indicated adequate statistical power to identify a clinically meaningful 15 percentage-point absolute difference in pCR rates.

## Results

### Clinicopathological features

In total, 209 patients who were treated for locally advanced resectable ESCC during the study period were eligible for enrolment in the study. There were 119 patients in the camrelizumab group and 90 in the tislelizumab group. Their clinicopathological characteristics are shown in **Table 1**. There was no significant between-group difference in any of the baseline characteristics, including age, sex, body mass index, smoking status, alcohol consumption, tumor location, tumor differentiation, clinical T stage, clinical N stage, clinical TNM stage, therapeutic regimen, surgical procedure performed, hypertension, or diabetes. However, more neoadjuvant treatment cycles were administered in the tislelizumab group than in the camrelizumab group.

**Table 1:** Patient baseline characteristics.

Variables	Camrelizumab (n=119)	Tislelizumab (n=90)	*P*-value
Age			1.00
≥65	61 (51.3%)	46 (51.1%)	
<65	58 (48.7%)	44 (48.9%)	
Gender			0.73
Male	91(76.5%)	65 (72.2%)	
Female	28 (23.5%)	25 (27.8%)	
BMI (kg/m^2^)			0.67
≥22.5	71 (59.7%)	51 (56.7%)	
<22.5	48 (40.3%)	39 (43.3%)	
Smoking			0.87
Yes	31 (26.1%)	22 (24.4%)	
No	88 (73.9%)	68 (75.6%)	
Alcohol Drinking			1.00
Yes	39 (32.8%)	29 (32.2%)	
No	80 (67.2%)	61 (67.8%)	
Tumor Location			0.12
Upper-thoracic	19 (16.0%)	16 (17.8%)	
Middle-thoracic	68 (57.1%)	39 (43.3%)	
Lower-thoracic	32 (26.9%)	35 (38.9%)	
Differentiation			0.81
Well	11 (9.2%)	7 (7.8%)	
Moderately/ poorly	108 (90.8%)	83 (92.2%)	
Clinical T Stage			0.06
2	15 (12.6%)	17 (18.9%)	
3	70 (58.8%)	59 (65.5%)	
4a	34 (28.6%)	14 (15.6%)	
Clinical N Stage			0.74
0	23 (19.3%)	22 (24.5%)	
1	76 (63.9%)	56 (62.2%)	
2	19 (16.0%)	12 (13.3%)	
3	1 (0.8%)	0 (0.0%)	
Clinical TNM Stage			0.15
II	32 (26.9%)	34 (37.8%)	
III	45 (37.8%)	34 (37.8%)	
IVA	42 (35.3%)	22 (24.4%)	
Therapeutic Regimen			0.84
Platinum+albumin-bound paclitaxel	103 (86.6%)	77 (85.6%)	
Platinum+docetaxel	16 (13.4%)	13 (14.4%)	
Neoadjuvant cycle			0.03
1	17 (14.3%)	9 (10.0%)	
2	63 (52.9%)	33 (36.7%)	
3	29 (24.4%)	35 (38.9%)	
4	10 (8.4%)	13 (14.4%)	
Surgical procedure			1.00
Ivor Lewis	14 (11.8%)	11 (12.2%)	
McKeown	105 (88.2%)	79 (87.8%)	
Hypertension			1.00
Yes	26 (21.8%)	20 (22.2%)	
No	93(78.2%)	70 (77.8%)	
Diabetes			1.00
Yes	17 (14.3%)	12 (13.3%)	
No	102 (85.7%)	78 (86.7%)	

### Outcomes of neoadjuvant treatment

The postoperative pathological results for the camrelizumab group indicated achievement of a pCR in 36 patients (30.3%), an MPR in 51 (42.9%), TRG 0 in 39 (32.8%), and pathological downstaging in 88 (73.9%) (**Table 2**). Similarly, in the tislelizumab group, 28 patients (31.1%) achieved a pCR, 40 (44.4%) achieved an MPR, 35 (38.9%) achieved TRG 0, and 61 (67.8%) achieved pathological downstaging. There was no statistically significant difference in the pathological response between the two groups.

**Table 2:** Pathological outcomes of neoadjuvant therapy.

Variables	Camrelizumab (n=119)	Tislelizumab (n=90)	*P*-value
PCR	36 (30.3%)	28 (31.1%)	1.00
MPR	51 (42.9%)	40 (44.4%)	0.89
Tumor regression grade			0.32
0	39 (32.8%)	35 (38.9%)	
1	14 (11.8%)	5 (5.6%)	
2	21 (17.6%)	20 (22.2%)	
3	45 (37.8%)	30 (33.3%)	
Pathologic downstaging	88 (73.9%)	61 (67.8%)	0.36

Subgroup analyses revealed that in the camrelizumab group, significantly higher pCR rates were observed among female patients, those with clinical N0 stage, clinical stage II disease, and docetaxel-treated individuals. Similarly, MPR rates were significantly elevated in female patients, clinical stage II cases, and docetaxel-treated subgroups within the camrelizumab cohort. In contrast, the tislelizumab group demonstrated distinct predictive patterns, with pCR rates showing significant associations with patient age, tumor location, and clinical N stage. Furthermore, MPR rates in the tislelizumab cohort were significantly correlated with tumor location and clinical N stage. (**Table 3**).

**Table 3:** Univariate analysis of subgroup.

Variables	Camrelizumab						Tislelizumab					
	PCR		*P*-value	MPR		*P*-value	PCR		*P*-value	MPR		*P*-value
	(-)	(+)		(-)	(+)		(-)	(-)		(-)	(+)	
Age			0.84			0.35			0.01			0.53
≥65	42 (51%)	19 (53%)		32 (47%)	29 (57%)		26 (42%)	20 (71%)		24 (48%)	22 (55%)	
<65	41 (49%)	17 (47%)		36 (53%)	22 (43%)		36 (58%)	8 (29%)		26 (52%)	18 (45%)	
Gender			0.02			0.02			0.13			0.48
Male	69 (82%)	22 (61%)		57 (84%)	33 (65%)		48 (77%)	17 (61%)		38 (76%)	27 (68%)	
Female	15 (18%)	14 (39%)		11 (16%)	18 (35%)		14 (23%)	11 (39%)		12 (24%)	13 (32%)	
BMI (kg/m^2^)			0.68			0.58			0.37			0.39
≥22.5	48 (58%)	23 (64%)		39 (57%)	32 (63%)		33 (53%)	18 (64%)		26 (52%)	25 (63%)	
<22.5	35 (42%)	13 (36%)		29 (43%)	19 (37%)		29 (47%)	10 (36%)		24 (48%)	15 (37%)	
Smoking			0.82			0.29			0.60			0.33
Yes	21 (25%)	10 (28%)		15 (22%)	16 (31%)		14 (23%)	8 (29%)		10 (20%)	12 (30%)	
No	62 (75%)	26 (72%)		53 (78%)	35 (69%)		48 (77%)	20 (71%)		40 (80%)	28 (70%)	
Alcohol Drinking			0.53			0.84			0.81			1.00
Yes	29 (35%)	10 (28%)		23 (34%)	16 (31%)		21 (34%)	8 (29%)		16 (32%)	13 (32%)	
No	54 (65%)	26 (72%)		45 (66%)	35 (69%)		41 (66%)	20 (71%)		34 (68%)	27 (68%)	
Tumor Location			0.13			0.74			<0.01			<0.01
Upper-thoracic	17 (21%)	2 (6%)		10 (15%)	9 (18%)		7 (11%)	9 (32%)		6 (12%)	10 (25%)	
Middle-thoracic	45 (54%)	23 (64%)		38 (56%)	30 (59%)		33 (53%)	6 (22%)		29 (58%)	10 (25%)	
Lower-thoracic	21 (25%)	11 (31%)		20 (29%)	12 (23%)		22 (36%)	13 (46%)		15 (30%)	20 (50%)	
Differentiation			1.00			1.00			1.00			1.00
Well	8 (10%)	3 (8%)		6 (9%)	5 (10%)		5 (8%)	2 (7%)		4 (8%)	3 (8%)	
Moderately/ poorly	75 (90%)	33 (92%)		62 (91%)	46 (90%)		57 (92%)	26 (93%)		46 (92%)	37 (92%)	
Clinical T Stage			0.29			0.21			0.80			0.90
2	9 (11%)	6 (17%)		6 (9%)	9 (18%)		13 (21%)	4 (14%)		10 (20%)	7 (18%)	
3	47 (57%)	23 (64%)		39 (57%)	31 (61%)		40 (64%)	19 (68%)		33 (66%)	26 (64%)	
4a	27 (32%)	7 (19%)		23 (34%)	11 (22%)		9 (15%)	5 (18%)		7 (14%)	7 (18%)	
Clinical N Stage			0.02			0.17			0.04			0.01
0	11 (13%)	12 (33%)		10 (15%)	13 (25%)		11 (18%)	11 (39%)		7 (14%)	15 (38%)	
≥1	72 (87%)	24 (67%)		58 (85%)	38 (75%)		51 (82%)	17 (61%)		43 (86%)	25 (62%)	
Clinical TNM Stage			<0.01			0.04			0.64			0.13
II	16 (19%)	16 (44%)		13 (19%)	19 (37%)		22 (35%)	12 (43%)		15 (30%)	19 (48%)	
III/ IVA	67 (81%)	20 (56%)		55 (81%)	32 (63%)		40 (65%)	16 (57%)		35 (70%)	21 (52%)	
Therapeutic Regimen			0.02			<0.01			0.53			0.55
Platinum+albumin-bound paclitaxel	76 (92%)	27 (75%)		64 (94%)	39 (76%)		54 (87%)	23 (82%)		44 (88%)	33 (82%)	
Platinum+docetaxel	7 (8%)	9 (25%)		4 (6%)	12 (24%)		8 (13%)	5 (18%)		6 (12%)	7 (18%)	
Neoadjuvant cycle			0.67			0.33			0.07			0.06
1/2	57 (69%)	23 (64%)		43 (63%)	37 (73%)		33 (53%)	9 (32%)		28 (56%)	14 (35%)	
¾	26 (31%)	13 (36%)		25 (37%)	14 (27%)		29 (47%)	19 (68%)		22 (44%)	26 (65%)	
Hypertension			0.09			0.39			1.00			0.80
Yes	22 (27%)	4 (11%)		19 (28%)	7 (14%)		14 (23%)	6 (21%)		12 (24%)	8 (20%)	
No	61 (73%)	32 (89%)		49 (72%)	44 (86%)		48 (77%)	22 (79%)		38 (76%)	32 (80%)	
Diabetes			0.08			1.00			1.00			1.00
Yes	10 (12%)	7 (19%)		10 (15%)	7 (14%)		8 (13%)	4 (14%)		7 (14%)	5 (13%)	
No	73 (88%)	29 (81%)		58 (85%)	44 (86%)		54 (87%)	24 (86%)		43 (86%)	35 (87%)	

### Safety and complications

The adverse reactions associated with neoadjuvant therapy are summarized in **Table 4**. The incidence rates of leukopenia, thrombocytopenia, immune-related thyroiditis, immune-related pneumonitis, and immune-related myocarditis was comparable between the two groups (all *P*>0.05). There was no statistically significant between-group difference in operation time, intraoperative blood loss, or the R0 resection rate (**Table 5**), or in the incidence rates of postoperative complications, including anastomotic leak, postoperative bleeding, thrombosis, pneumonia, respiratory failure, arrhythmia, and mortality within 30 days. However, the rate of unplanned postoperative transfer to the intensive care unit (ICU) was lower in the tislelizumab group than in the camrelizumab group. Sensitivity analyses excluding elderly patients (>75 years) and prolonged surgeries (>10 hours) confirmed the robustness of our primary finding, with tislelizumab group maintaining significantly reduced ICU transfer rates (**Supplementary Tables 1**, **2**). Multivariate logistic regression analysis identified clinical TNM stage, PD-1 inhibitor type, postoperative pneumonia, and respiratory failure as risk factors for unplanned postoperative ICU transfer (**Supplementary Table 3**).

**Table 4:** The adverse events of neoadjuvant therapy.

Variables	Camrelizumab (n=119)	Tislelizumab (n=90)	*P*-value
Leukopenia	30 (25.2%)	21 (23.3%)	0.87
Thrombocytopenia	10 (8.4%)	8 (8.9%)	1.00
Immune-related thyroiditis	10 (8.4%)	10 (11.1%)	0.64
Immune-related pneumonitis	2 (1.7%)	0 (0.0%)	0.51
Immune-related myocarditis	1 (0.8%)	0 (0.0%)	1.00

**Table 5:** The perioperative outcomes of esophagectomy after neoadjuvant therapy.

Variables	Camrelizumab (n=119)	Tislelizumab (n=90)	*P*-value
Time from last neoadjuvant treatment to surgery (days)			
Median (IQR)	36 (32, 38)	37 (33, 39)	0.14
Operation time (min)			0.95
Median (IQR)	320 (290, 400)	330 (300, 380)	
Intraoperative blood loss (ml)			0.16
Median (IQR)	150 (100, 200)	100 (100, 200)	
Margin status			0.45
R0	111 (93.3%)	81 (90.0%)	
R1	8 (6.7%)	9 (10.0%)	
Anastomotic leak	15 (12.6%)	5 (5.6%)	0.10
Postoperative bleeding	3 (2.5%)	0 (0.0%)	0.26
Thrombosis	4 (3.4%)	1 (1.1%)	0.39
Pneumonia	30 (25.2%)	23 (25.6%)	1.00
Respiratory failure	8 (6.7%)	12 (13.3%)	0.15
Arrhythmia	33 (27.7%)	26 (28.9%)	0.88
Unplanned transfer to ICU	44 (37.0%)	21 (23.3%)	0.04
Mortality within 30 days	1 (0.8%)	0 (0.0%)	1.00

## Discussion

At this time, standard neoadjuvant therapy for locally advanced ESCC continues to consist of chemotherapy or chemoradiotherapy. However, the advent of immunotherapy, immune checkpoint inhibitors in particular, marks a significant advance in the treatment of advanced esophageal cancer. Multiple studies indicate that neoadjuvant chemotherapy combined with immunotherapy offers a new treatment opportunity for locally advanced ESCC (15–18). Most surgeons believe that esophagectomy is no more difficult after neoadjuvant chemoimmunotherapy than after neoadjuvant chemoradiotherapy. Yu et al. demonstrated that neoadjuvant chemoimmunotherapy in patients with ESCC was associated with R0 resection and pCR rates that were comparable with those of neoadjuvant chemoradiotherapy and that the prognosis appeared to be better after neoadjuvant chemoimmunotherapy (19). Wang et al. also found neoadjuvant chemoimmunotherapy to be a safe and feasible strategy for locally advanced ESCC (20). Among the various immunotherapeutic agents currently used for ESCC, only camrelizumab has been shown in a Phase III clinical study to be more effective than chemotherapy alone when used in combination with chemotherapy in the neoadjuvant setting (12). Therefore, selecting the optimal neoadjuvant immunotherapy regimen for locally advanced ESCC is a challenge that still needs to be addressed.

In this study, we compared the perioperative efficacy and safety of neoadjuvant chemotherapy combined with camrelizumab with that of neoadjuvant chemotherapy combined with tislelizumab for locally advanced ESCC. Although the tislelizumab group had received more treatment cycles at baseline, both groups had comparable pCR, MPR, and tumor downstaging rates. The two groups also performed similarly well in terms of safety. However, the rate of unplanned postoperative transfer to the ICU was lower in the tislelizumab group.

Patients who achieve a pCR after neoadjuvant therapy generally have a favorable long-term prognosis despite the risk of recurrence (21, 22). Therefore, the pCR and MPR are currently regarded as reliable surrogate endpoints for assessing the effectiveness of neoadjuvant therapy. In this study, the pCR rate for camrelizumab was 30.3% and that for tislelizumab was 31.1%. Compared with the 9% pCR rate for standard neoadjuvant chemotherapy, our outcomes were better and numerically in line with the 32% seen after neoadjuvant chemoradiotherapy for locally advanced ESCC in a meta-analysis (23). Subgroup analyses demonstrated differential predictive factors for pCR and MPR in the camrelizumab and tislelizumab cohorts. While certain variables reached significance in univariate analysis, none remained independently predictive in multivariate regression models. In ESCC, PD-L1 expression levels—an important biomarker of immune checkpoint inhibitor efficacy—are integral to risk stratification and treatment personalization. Both the ESCORT-NEO/NCCES01 and TD-NICE datasets demonstrated that elevated PD-L1 expression was significantly associated with improved pCR rates (12, 14). As a retrospective study, our analysis lacked PD-L1 expression data, which may explain the limited predictive value for pCR and MPR observed in subgroup analyses. At present, there are no clinical tools that can reliably predict the pCR in patients with ESCC (24). Several studies have found that CT radiomics can predict pCR status after neoadjuvant therapy for ESCC (25–27). Feng et al. also reported that the pretreatment integrative inflammatory and nutritional score is an independent predictor of pCR in patients with ESCC treated using chemoimmunotherapy (28). Prospective multicenter studies incorporating novel biomarkers and advanced machine learning algorithms are warranted to establish a robust predictive model for pCR following neoadjuvant therapy in ESCC patients. R0 resection status is important when assessing the success of surgery for ESCC because it correlates with patient outcomes and is a measure of effective treatment (29). In our study, there was no notable difference in the R0 resection rate between the two groups. The R0 resection rate was high in both the camrelizumab and tislelizumab groups (93.3% vs 90.0%). Perioperative morbidity and mortality are the primary concerns in surgery following neoadjuvant therapy. A recent retrospective analysis of 133 patients with ESCC demonstrated favorable perioperative safety profiles for neoadjuvant chemoimmunotherapy. While subgroup analyses indicated comparable safety outcomes across different immunotherapeutic agents, the limited sample size in each treatment subgroup warrants cautious interpretation of these findings (30). Zhang et al. reported that camrelizumab plus neoadjuvant chemotherapy yielded superior surgical outcomes versus chemoradiotherapy in ESCC, with shorter operations, less bleeding, and reduced ICU stays (4). The safety of tislelizumab during the perioperative period has been confirmed in other large-scale clinical studies (31, 32). In a retrospective study of gastric cancer, tislelizumab did not increase the incidence of postoperative complications (33). We did not detect a statistically significant difference in the incidence of neoadjuvant therapy-related adverse events between our two groups. In terms of postoperative complications, the proportion of patients who underwent unplanned transfer to the ICU after surgery was lower in the tislelizumab group. While ESCORT-NEO reported comparable perioperative morbidity between camrelizumab and chemotherapy arms, our observed ICU transfer reduction with tislelizumab may reflect differential immune-related toxicity profiles. Several immunologically plausible explanations merit consideration. First, as a humanized IgG4 anti-PD-1 antibody with engineered Fc receptor binding, tislelizumab may cause less cytokine-mediated toxicity compared to other checkpoint inhibitors. Second, its preserved T-regulatory cell function might maintain immunological homeostasis during surgical stress. Third, the observed effect could reflect reduced subclinical cardiotoxicity or pulmonary inflammation (34, 35). The observed discrepancy may be also attributable to several factors, including patient selection bias, heterogeneity in ICU admission criteria across clinical scenarios, and variations in postoperative management protocols within our institution. Prospective trials incorporating serial immune monitoring are needed to test these hypotheses.

This study had some limitations. First, it had a single-center retrospective real-world design with a small sample size, which meant that selection bias was unavoidable. Our study remains underpowered to detect rare outcomes such as myocarditis and mortality due to their low incidence rates. Future multi-center prospective head-to-head comparative studies would provide more robust evidence to address this issue. Second, this study was limited to comparing perioperative outcomes, with no data available on OS or DFS. Future studies with long-term follow-up are warranted to evaluate potential differences in survival outcomes. Third, there were no data on PD-L1 expression, so it was not possible to assess the relationship between PD-L1 expression and the response to neoadjuvant therapy in detail. The precise molecular mechanisms responsible for the differential therapeutic effects remain to be elucidated.

In conclusion, this real-world analysis has demonstrated that neoadjuvant chemotherapy plus tislelizumab is safe and feasible in patients with locally advanced ESCC, and has satisfactory pCR and MPR rates. However, long-term survival requires further investigation. Chemoimmunotherapy that includes tislelizumab may become a neoadjuvant treatment option for locally advanced ESCC in the future.

## Data Availability

The raw data supporting the conclusions of this article will be made available by the authors, without undue reservation.
