# Supplementary material for: Perioperative outcomes of neoadjuvant chemotherapy plus camrelizumab versus neoadjuvant chemotherapy plus tislelizumab for locally advanced esophageal squamous cell cancer: a real-world retrospective study
Source: Front Immunol. 2025 Aug 21;16:1544739. doi: 10.3389/fimmu.2025.1544739 (PMC12408292; doi:10.3389/fimmu.2025.1544739)
Supplement: Supplementary file 1 [file Table1.docx]

Supplementary Table 1 Sensitivity analysis after excluding patients older than 75 years

| Variables | Camrelizumab  (n=114) | Tislelizumab  (n=84) | *P*-value |
| --- | --- | --- | --- |
| Time from last neoadjuvant treatment to surgery (days) |  |  | 0.16 |
| Median (IQR) | 35.5 (32, 38) | 37 (33, 39) |  |
| Operation time (min) |  |  | 0.95 |
| Median (IQR) | 310 (290, 390) | 330 (300, 380) |  |
| Intraoperative blood loss (ml) |  |  | 0.08 |
| Median (IQR) | 150 (100, 200) | 100 (100, 150) |  |
| Margin status |  |  | 0.44 |
| R0 | 106 (93.0%) | 75 (89.3%) |  |
| R1 | 8 (7.0%) | 9 (10.7%) |  |
| Anastomotic leak | 15 (13.2%) | 4 (4.8%) | 0.05 |
| Postoperative bleeding | 3 (2.6%) | 0 (0.0%) | 0.26 |
| Thrombosis | 4 (3.5%) | 1 (1.2%) | 0.40 |
| Pneumonia | 29 (25.4%) | 22 (26.2%) | 1.00 |
| Respiratory failure | 8 (7.0%) | 12 (14.3%) | 0.10 |
| Arrhythmia | 32 (28.1%) | 24 (28.6%) | 1.00 |
| Unplanned transfer to ICU | 44 (38.6%) | 20 (23.8%) | 0.03 |
| Mortality within 30 days | 1 (0.9%) | 0 (0.0%) | 1.00 |

Supplementary Table 2 Sensitivity analysis after excluding patients with operative times exceeding 10 hours

| Variables | Camrelizumab  (n=116) | Tislelizumab  (n=90) | *P*-value |
| --- | --- | --- | --- |
| Time from last neoadjuvant treatment to surgery (days) |  |  | 0.14 |
| Median (IQR) | 36 (32, 38) | 37 (33, 39) |  |
| Operation time (min) |  |  | 0.71 |
| Median (IQR) | 315 (290, 390) | 330 (300, 380) |  |
| Intraoperative blood loss (ml) |  |  | 0.18 |
| Median (IQR) | 150 (100, 200) | 100 (100, 200) |  |
| Margin status |  |  | 0.45 |
| R0 | 108 (93.1%) | 81 (90.0%) |  |
| R1 | 8 (6.9%) | 9 (10.0%) |  |
| Anastomotic leak | 15 (12.9%) | 4 (5.6%) | 0.09 |
| Postoperative bleeding | 2 (1.7%) | 0 (0.0%) | 0.51 |
| Thrombosis | 4 (3.4%) | 1 (1.1%) | 0.40 |
| Pneumonia | 29 (25.0%) | 23 (25.6%) | 1.00 |
| Respiratory failure | 7 (6.0%) | 12 (13.3%) | 0.09 |
| Arrhythmia | 32 (27.6%) | 26 (28.9%) | 0.88 |
| Unplanned transfer to ICU | 43 (37.1%) | 21 (23.3%) | 0.04 |
| Mortality within 30 days | 1 (0.9%) | 0 (0.0%) | 1.00 |

Supplementary Table 3 Univariate and multivariate analysis of unplanned transfer to ICU

| Variables | Univariate analysis | | | Multivariable analysis |
| --- | --- | --- | --- | --- |
|  | Unplanned transfer to ICU | | *P*-value | *P*-value |
|  | NO | YSE |  |  |
| Age |  |  | 0.30 | - |
| ≥65 | 74 (51.4%) | 28 (43.1%) |  |  |
| ＜65 | 70 (48.6%) | 37 (56.9%) |  |  |
| BMI (kg/m^2^) |  |  | 0.37 | - |
| ≥22.5 | 63 (43.7%) | 24 (36.9%) |  |  |
| ＜22.5 | 81 (56.3%) | 41 (63.1%) |  |  |
| Smoking |  |  | 0.87 | - |
| Yes | 31 (26.1%) | 22 (24.4%) |  |  |
| No | 88 (73.9%) | 68 (75.6%) |  |  |
| Alcohol Drinking |  |  | 0.26 | - |
| Yes | 43 (29.9%) | 25 (38.5%) |  |  |
| No | 101 (70.1%) | 40 (61.5%) |  |  |
| Hypertension |  |  | 0.37 | - |
| Yes | 29 (20.1%) | 17 (26.2%) |  |  |
| No | 115 (79.9%) | 48 (73.8%) |  |  |
| Diabetes |  |  | 0.09 | - |
| Yes | 24 (16.7%) | 5 (7.7%) |  |  |
| No | 120 (83.3%) | 60 (92.3%) |  |  |
| Tumor Location |  |  | 0.94 | - |
| Upper-thoracic | 25 (17.4%) | 10 (15.4%) |  |  |
| Middle-thoracic | 74 (51.4%) | 33 (50.8%) |  |  |
| Lower-thoracic | 45 (31.2%) | 22 (33.8%) |  |  |
| Differentiation |  |  | 0.44 | - |
| Well | 11 (7.6%) | 7 (10.8%) |  |  |
| Moderately/ poorly | 133 (92.4%) | 58 (89.2%) |  |  |
| Clinical TNM Stage |  |  | 0.03 | 0.02 |
| II | 43 (29.9%) | 23 (35.4%) |  |  |
| III | 49 (34.0%) | 30 (46.2%) |  |  |
| IVA | 52 (36.1%) | 12 (18.5%) |  |  |
| Therapeutic regimen |  |  | 0.04 | <0.01 |
| Camrelizumab | 75 (52.1%) | 44 (67.7%) |  |  |
| Tislelizumab | 69 (47.9%) | 21 (32.3%) |  |  |
| Neoadjuvant cycle |  |  | 0.58 | - |
| 1 | 19 (13.2%) | 7 (10.8%) |  |  |
| 2 | 68 (47.2%) | 28 (43.1%) |  |  |
| 3 | 44 (30.6%) | 20 (30.8%) |  |  |
| 4 | 13 (9.0%) | 10 (15.4%) |  |  |
| Surgical procedure |  |  | 0.36 | - |
| Ivor Lewis | 15 (10.4%) | 10 (15.4%) |  |  |
| McKeown | 129 (89.6%) | 55 (84.6%) |  |  |
| Operation time (min) |  |  | 0.66 | - |
| Median (IQR) | 335 (300, 395) | 310 (300, 380) |  |  |
| Intraoperative blood loss (ml) |  |  | 0.20 | - |
| Median (IQR) | 150 (100, 200) | 150 (100, 200) |  |  |
| Margin status |  |  | 1.00 | - |
| R0 | 132 (91.7%) | 5 (7.7%) |  |  |
| R1 | 12 (8.3%) | 60 (92.3%) |  |  |
| Anastomotic leak |  |  | 0.07 | - |
| Yes | 10 (6.9%) | 10 (15.4%) |  |  |
| No | 134 (93.1%) | 55 (84.6%) |  |  |
| Postoperative bleeding |  |  | 0.23 | - |
| Yes | 1 (0.7%) | 2 (3.1%) |  |  |
| No | 143 (99.3%) | 63 (96.9%) |  |  |
| Thrombosis |  |  | 0.65 | - |
| Yes | 3 (2.1%) | 2 (3.1%) |  |  |
| No | 141 (97.9%) | 63 (96.9%) |  |  |
| Pneumonia |  |  | <0.01 | <0.01 |
| Yes | 22 (15.3%) | 31 (47.7%) |  |  |
| No | 122 (84.7%) | 34 (52.3%) |  |  |
| Respiratory failure |  |  | <0.01 | <0.01 |
| Yes | 1 (0.7%) | 19 (29.2%) |  |  |
| No | 143 (99.3%) | 46 (70.8%) |  |  |
| Arrhythmia |  |  | 0.01 | - |
| Yes | 33 (22.9%) | 26 (40.0%) |  |  |
| No | 111 (77.1%) | 39 (60.0%) |  |  |

Supplementary Table 4 Comparison of perioperative findings across our research and the ESCORT-NEO/TD-NICE studies

| Variables |  |  | ESCORT-NEO | | | TD-NICE |
| --- | --- | --- | --- | --- | --- | --- |
|  | Camrelizumab  (n=116) | Tislelizumab  (n=90) | Cam+nab-TP (n=114) | Cam+TP  (n=116) | TP  (n=103) | (n=36) |
| Operation time |  |  |  |  |  |  |
| Median (IQR) | 315 min  (290, 390) | 330 min  (300, 380) | 4.3 h  (2.6–8.9) | 4.2 h  (2.8–7.2) | 4.2 h  (2.9–10.8) | - |
| Intraoperative blood loss (ml) |  |  |  |  |  |  |
| Median (IQR) | 150  (100, 200) | 100  (100, 200) | - | - | - | 214 |
| Margin status |  |  |  |  |  |  |
| R0 | 108 (93.1%) | 81 (90.0%) | 113 (99.1%) | 111 (95.7%) | 95 (92.2%) | 29 (80.5%) |
| R1 | 8 (6.9%) | 9 (10.0%) | 1 (0.9%) | 4 (3.4%) | 6 (5.8%) | 6 (16.7%) |
| Anastomotic leak | 15 (12.9%) | 4 (5.6%) | 3 (2.6%) | 5 (4.3%) | 6 (5.8%) | 2 (5.6) |
| Postoperative bleeding | 2 (1.7%) | 0 (0.0%) | - | - | - | - |
| Thrombosis | 4 (3.4%) | 1 (1.1%) | - | - | - | - |
| Pneumonia | 29 (25.0%) | 23 (25.6%) | 12 (10.5%) | 21 (18.1%) | 15 (14.6%) | 14 (38.9) |
| Respiratory failure | 7 (6.0%) | 12 (13.3%) | 1 (0.9%) | 0 | 1 (1.0%) | - |
| Arrhythmia | 32 (27.6%) | 26 (28.9%) | 7 (6.1%) | 2 (1.7%) | 3 (2.9%) | 2 (5.6) |
| Unplanned transfer to ICU | 43 (37.1%) | 21 (23.3%) | - | - | - | - |
| Mortality within 30 days | 1 (0.9%) | 0 (0.0%) | 1 (0.9%) | 2 (1.7%) | 1 (1.0%) | 1 (2.8) |
